# Supplementary material for: Explainable depth-wise and channel-wise fusion models for multi-class skin lesion classification
Source: PLoS One. 2026 Jan 22;21(1):e0340901. doi: 10.1371/journal.pone.0340901 (PMC12826527; doi:10.1371/journal.pone.0340901)
Supplement: Attachment — Submitted filename: Response to Reviewers.pdf [file pone.0340901.s002.pdf]

► Response Letter for “PONE-D-25-11481”

September 07, 2025

Dear Academic Editor Lin Xu,

We always hope you are fine and doing well.

We thank you for the opportunity to revise and resubmit our manuscript "**Explainable Depth-Wise and Channel-Wise Fusion Models for Multi-Class Skin Lesion Classification**" (PONE-D-25-11481). We appreciate the constructive comments and suggestions from you and the reviewers, which have helped us improve the quality of our work. We have now thoroughly revised the manuscript and have carefully addressed each suggestion point-by-point below. For your convenience, all changes have been highlighted in the “Revised Manuscript with Track Changes” file.

Thanks!

<table>
<tr><th colspan="2">Journal Requirements</th></tr>
<tr><td rowspan="2">1.</td><td>1. Please ensure that your manuscript meets PLOS ONE’s style requirements, including those for file naming. The PLOS ONE style templates can be found at https://journals.plos.org/plosone/s/file?id=wjVg/PLOSOne_formatting_sample_main_body.pdf and https://journals.plos.org/plosone/s/file?id=ba62/PLOSOne_formatting_sample_title_authors_affiliations.pdf</td></tr>
<tr><td>Response:

We thank the editorial office for this reminder. We have taken several steps to ensure the revised manuscript fully complies with PLOS ONE’s formatting and style guidelines.

Specifically, we have downloaded and utilized the official PLOS ONE LaTeX template from the journal’s website for the revised version. We have also carefully reviewed the provided PDF templates for the main body and title page to ensure all sections, including affiliations, headings, figure captions, figure files, and reference formatting, align with the journal’s requirements. Besides, we have processed all figure files for our manuscript using the PACE tool as requested.

Furthermore, we confirm that upon resubmission, all files (including the “Response to Reviewers”, “Revised Manuscript with Track Changes”, and “Manuscript” files) will be named according to the specified conventions. We are confident that the revised submission now meets all PLOS ONE style requirements.</td></tr>
<tr><td>2.</td><td>Please note that PLOS ONE has specific guidelines on code sharing for submissions in which author-generated code underpins the findings in the manuscript. In these cases, we expect all author-generated code to be made available without restrictions upon publication of the work.

Please review our guidelines at https://journals.plos.org/plosone/s/materials-and-software-sharing#loc-sharing-code</td></tr>
</table>

	and ensure that your code is shared in a way that follows best practice and facilitates reproducibility and reuse.
	**Response:**<br>• **Data availability statement**: Page [39], lines [933-935]<br>• GitHub at (https://github.com/Radhwan-Saleh/SkinCancerFusionModel)<br><br>Thank you for the guidance on PLOS ONE's code sharing policy. We have prepared a public repository containing the author-generated code used to produce the findings presented in our manuscript. The repository includes the model implementations, training and evaluation scripts, a detailed README file with instructions for reproduction, a list of dependencies, and a permissive open-source (MIT) license.<br><br>Furthermore, we have revised the "**Data Availability Statement**", which provides a direct link to the public repository.<br><br>Revised **Data availability statement**:<br><br>"The dataset used in this study is publicly available. All source code is available on GitHub at (https://github.com/Radhwan-Saleh/SkinCancerFusionModel)."
**3.**	Thank you for stating the following **financial disclosure**:<br><br>"This work was supported by the National Research Foundation of Korea (NRF) grant funded by the Korean government (MSIT) (No. RS-2023-00256517)."<br><br>Please state what role the funders took in the study. If the funders had no role, please state: "The funders had no role in study design, data collection and analysis, decision to publish, or preparation of the manuscript." If this statement is not correct you must amend it as needed.<br><br>Please include this amended Role of Funder statement in your cover letter; we will change the online submission form on your behalf.
	**Response:**<br><br>Thank you for the instruction. We have an important update regarding our funding situation, the full details of which are provided in our cover letter. The funding originally declared is no longer available, and the manuscript has been revised accordingly. Our updated and final financial disclosure statements are now as follows:<br><br>• **Financial Disclosure:** The authors received no specific funding for this work.<br><br>As no specific funding was received for this study, the "Role of Funder" statement is not applicable. We have noted this in our cover letter for the editorial team.

**4.**	Thank you for stating the following in the **Acknowledgments Section** of your manuscript:<br><br>"This work was supported by the National Research Foundation of Korea (NRF) grant funded by the Korean government (MSIT) (No. RS-2023-00256517). The authors also acknowledge the support of the Scientific Research Projects (BAP, Project Number: 3681) and the Kocaeli University Software Technologies Research (STAR) Laboratory, which facilitated the completion of this study."<br><br>We note that you have provided funding information that is not currently declared in your Funding Statement. However, funding information should not appear in the Acknowledgments section or other areas of your manuscript. We will only publish funding information present in the Funding Statement section of the online submission form.<br><br>Please remove any funding-related text from the manuscript and let us know how you would like to update your Funding Statement. Currently, your Funding Statement reads as follows:<br><br>"This work was supported by the National Research Foundation of Korea (NRF) grant funded by the Korean government (MSIT) (No. RS-2023-00256517)."<br><br>Please include your amended statements within your cover letter; we will change the online submission form on your behalf.
	**Response:**<br><br>We confirm that we have fully complied with this requirement. All funding-related text has been removed from the manuscript. To ensure clarity and full adherence to journal policy, the entire Acknowledgments section has been removed in this revised version.
**5.**	If the reviewer comments include a recommendation to cite specific previously published works, please review and evaluate these publications to determine whether they are relevant and should be cited. There is no requirement to cite these works unless the editor has indicated otherwise.
	**Response:**<br><br>We acknowledge the journal's policy regarding recommended citations. We confirm that no specific publications were recommended for citation.
**6.**	Please review your reference list to ensure that it is complete and correct. If you have cited papers that have been retracted, please include the rationale for doing so in the manuscript text, or remove these references and replace them with relevant current references. Any changes to the reference list should be mentioned in the rebuttal letter that accompanies your revised manuscript. If you need to cite a retracted article, indicate the article's retracted status in the References list and also include a citation and full reference for the retraction notice.
	**Response:**<br><br>We thank the editorial office for this important instruction. We have performed a thorough review of our reference list for accuracy and completeness. To specifically check for retractions, we cross-referenced our entire bibliography against the Retraction Watch database, using their public dataset generated on August 22, 2025, and can confirm that none of our cited works have been retracted.

Reviewer 1	
	The study addresses a critical gap in skin cancer diagnostics by integrating XAI with deep learning models. A few constructive suggestions about this paper.
	**Response:**<br>Thank you for your insightful and constructive review. We appreciate your recognition of our work's significance and have carefully addressed each of your comments in the revised manuscript.<br>In the following, we provide point-by-point responses and outline the corresponding revisions made to improve the clarity, rigor, and overall quality of the paper. We believe these changes strengthen the manuscript and more clearly highlight its contributions to medical image analysis.
**1.**	No discussion on how the proposed models would integrate into dermatology workflows.
	**Response:**<br>• Page [37], lines [861-888]<br>We thank you for this relevant point. We agree that discussing the practical clinical utility of our proposed models is essential to framing the impact of our work.<br>To address this, we have added a new, dedicated subsection titled "**Integration into Clinical Workflow**" This section now elaborates on how our system could be deployed as a computer-aided diagnosis (CADx) tool, detailing its potential roles in both direct clinical decision-support, leveraging the dual XAI outputs as an interpretable, and in teledermatology triage to help prioritize patient care.
**2.**	The manuscript claims "random augmentation" but lacks specifics on transformation parameters. Critical details like whether augmentation preserved lesion morphology are missing.
	**Response:**<br>• Pages [15-16], lines [331-356]<br>Thank you for this critical comment regarding the need for methodological transparency and reproducibility. We have now substantially revised the "**Data augmentation**" section to address this thoroughly.<br>In the revised section, we now specify the exact transformations and their corresponding parameters used in our study, including horizontal and vertical flips (each with 50% probability), rotations (within a range of ±20 degrees), brightness adjustments (up to ±0.2) and contrast adjustments (up to ±0.3), and Gaussian blur (with a sigma limit of 0.1 to 1.0).<br>Furthermore, we explicitly address the point about preserving lesion morphology. We now state that these conservative parameters were deliberately selected to mimic realistic clinical variations (e.g., in lighting and orientation) without distorting diagnostically critical features. Most importantly, we have added a statement confirming that we visually inspected the augmented samples to ensure their clinical validity and the integrity of the lesion characteristics.

**3.**	Without linking SHAP values to dermatological features, interpretability claims are incomplete.
	**Response:**<br>• Pages [34-37], lines [787-860]<br>Thank you for this superb point, which gets to the very core of meaningful explainability in a clinical context. To rectify this, we have significantly revised and expanded the "**SHapley Additive exPlanations (SHAP)**" subsection. The analysis is no longer just a summary of heatmap quality but is now a direct interpretation of the model's reasoning in a clinical context.<br>The key enhancements in the revised section are:<br>• Clinical Framework: We now explicitly introduce the ABCD rule as a clinical framework to provide a common language for interpreting the model's feature importance.<br>• Detailed Case Studies: We perform a series of in-depth case studies using examples from Figure 12. This includes a comparative analysis of how all our fusion models interpret.<br>• Comparative Analysis: The entire subsection is now framed as a comparative analysis of fusion strategies, discussing the trade-offs in interpretability that each architectural choice entails. This directly aligns with the investigative spirit of our manuscript.
**4.**	Specify whether images were preprocessed (e.g., color normalization).
	**Response:**<br>• Page [14], lines [305-314]<br>We thank the reviewer for raising this point, which highlights the need for a more detailed methodological description. While we had summarized the key preprocessing steps in the overview diagram of our framework (Figure 1), we acknowledge that a thorough explanation of these steps was not adequately detailed in the main body of the manuscript, which is essential for transparency and reproducibility.<br>To rectify this, we have now added several new paragraphs to the "**Dataset and preprocessing**" subsection. This text provides a comprehensive and explicit account of our entire preprocessing pipeline. The steps, now clearly detailed, include:<br>• Resizing: We specify that all images were uniformly resized from their original resolution to 224x224 pixels to meet the input requirements of the backbone models.<br>• Intensity Normalization: We clarify the exact method of color normalization used, which was to scale the pixel values of each channel from their original integer range to a [0.0, 1.0] floating-point range by dividing by 255.
**5.**	Why do the authors use HAM10000 instead of ISIC2023?
	**Response:**<br>Thank you for this question regarding dataset selection. While the ISIC 2023 challenge dataset is not available on the official ISIC Archive, our analysis of the other recent challenges (2020, 2021, 2022, and 2024) confirms that HAM10000 was the most appropriate choice for our study.

	The primary reason is task alignment. Our research objective is specifically multi-class classification across seven distinct lesion types from a single image. The recent ISIC challenges, in contrast, have consistently focused on binary classification (e.g., malignant vs. benign) or different modalities entirely (e.g., multimodal data, longitudinal 3D imaging). These tasks are fundamentally different from our research question.<br><br>Consequently, HAM10000 is the most widely-used benchmark for the multi-class classification task we address, making it essential for comparing our results against other work. This choice was necessary to fairly benchmark our fusion models against the field's leading methods and validate our findings.

**Reviewer 2**	
	This paper focuses on developing and rigorously evaluating novel explainable AI (XAI) special and channel fusion models for multi-class skin lesion classification. The research is of great practical significance, with sufficient workload and convincing experiments.
	**Response:**<br><br>Thank you for your positive and we appreciate your recognition of the importance of our proposed framework for skin cancer detection and classification. We also value your constructive suggestions for further improvements. We have carefully considered each of your comments and have made revisions accordingly to enhance the clarity, depth, and rigor of our work. Below, we provide detailed responses to your suggestions and the corresponding updates made in the manuscript.
**1.**	From the perspective of the paper, improvements and innovations should be strengthened in the abstract section, focusing on how to solve problems.
	**Response:**<br>• Pages [1-2]<br><br>We thank the reviewer for this insightful feedback. Upon review, we recognized that while our abstract accurately described the components of our study, it did not effectively communicate the central innovation or the problem-solving narrative.<br><br>To address this, we have completely rewritten the abstract to be more focused and impactful. The new version has been restructured to tell a clearer story, as follows:<br>• It now explicitly frames the research around solving the dual challenge of achieving both high accuracy and clinical transparency in dermatological AI.<br>• It introduces our core innovation upfront: the systematic investigation of fusing the local feature expertise of CNNs with the global contextual understanding of Vision Transformers (ViTs) as our specific solution to this problem.<br>• The abstract now more clearly links our quantitative results (90% F1-score) to solving the accuracy problem and our XAI analysis (demonstrating alignment with clinical reasoning) to solving the transparency problem.

**2.**	The paper should include more recent research findings for discussion, such as 2024-2025. If the author can solve the above problems, we think the paper is excellent.
	**Response:**<br>Thank you for this suggestion. We have conducted a thorough literature search and have identified four highly relevant, state-of-the-art studies published in 2024 and 2025. We have now carefully integrated these recent findings into the manuscript to provide a more comprehensive and up-to-date discussion.<br>These significant updates have been made in two key sections of the paper:<br>1- In the "**Related Works**" Section, Pages [8-9, 10-11], lines [194-213] and lines [265-275].<br>2- In the "**Comparison with literature**" Section, Table 5, Page [32].
