# Supplementary material for: Explainable depth-wise and channel-wise fusion models for multi-class skin lesion classification
Source: PLoS One. 2026 Jan 22;21(1):e0340901. doi: 10.1371/journal.pone.0340901 (PMC12826527; doi:10.1371/journal.pone.0340901)
Supplement: Attachment — Submitted filename: Response_to_Reviewers_auresp_2.pdf [file pone.0340901.s003.pdf]

► Response Letter for “PONE-D-25-11481R1”

December 16, 2025

Dear Academic Editor Lin Xu,

We always hope you are fine and doing well.

We thank you for the opportunity to revise and resubmit our manuscript "**Explainable Depth-Wise and Channel-Wise Fusion Models for Multi-Class Skin Lesion Classification**" (PONE-D-25-11481R1). We appreciate the constructive comments and suggestions from you and the reviewers, which have helped us improve the quality of our work. We have now thoroughly revised the manuscript and carefully addressed each suggestion point by point below. For your convenience, all changes have been highlighted in the “Revised Manuscript with Track Changes” file.

Thanks!

Journal Requirements	
**1.**	If the reviewer comments include a recommendation to cite specific previously published works, please review and evaluate these publications to determine whether they are relevant and should be cited. There is no requirement to cite these works unless the editor has indicated otherwise.
	**Response:**<br>We thank the Editor for this guidance. In accordance with the Journal Requirements, we have carefully evaluated the list of publications suggested during the review process. We applied a systematic evaluation strategy to determine their scientific relevance to our study's scope, specifically prioritizing works that address hybrid architectures and explainable AI in medical imaging. Consequently, we have incorporated only those references that directly enhance the quality and contextual depth of our manuscript, ensuring that all citations are scientifically justified.

Reviewer #1	
	Dear authors, after careful review, I have no additional concerns regarding your submission at this time.
	**Response:**<br>Thank you for your insightful and constructive review.

Reviewer #3	
	The paper presents a comprehensive study on explainable multi-class skin lesion classification by designing six fusion-based deep learning models that combine the local feature extraction strength of CNNs with the global contextual capabilities of Vision Transformers (ViTs). Using the challenging 7-class HAM10000 dataset, the authors rigorously evaluate depth-wise and channel-wise fusion strategies and achieve a strong weighted Precision, Recall, and F1 of 90%. A key contribution of this work is its emphasis on clinical interpretability, where the authors employ Grad-CAM and SHAP to show that the best-performing fusion models focus on medically meaningful attributes such as border irregularity and color variegation—aligning with dermatological reasoning. Overall, the paper offers an accurate, transparent, and clinically aligned AI framework, highlighting how fusion architecture choices directly influence both performance and explainability in automated skin cancer diagnosis.
	**Response:**<br>Thank you for your positive and we appreciate your recognition of the importance of our proposed framework for skin cancer classification. We also value your constructive suggestions for further improvements. We have carefully considered each of your comments and have made revisions accordingly to enhance the clarity, depth, and rigor of our work. Below, we provide detailed responses to your suggestions and the corresponding updates made in the manuscript.
**1.**	Transparency and Reproducibility: The authors should provide full open-source code, pretrained models, and data preprocessing scripts in a publicly accessible repository. Including reproducible. Although code is included, it's recommended that a Jupyter notebooks for training, evaluation, and ablation would demonstrate commitment to transparency.
	**Response:**<br>We thank the reviewer for emphasizing the importance of transparency and reproducibility. We confirm that the GitHub link provided in the manuscript currently contains the open-source code required to verify and control our work, including the model architecture, Grad-CAM, and SHAP analysis scripts.<br>Regarding the pretrained models, weights, and comprehensive notebooks, we have organized these in a private mode, and they will be made fully public immediately upon the manuscript's acceptance at the same repository link provided in the article.
**2.**	Comprehensive Dataset and Method Details. Expand dataset descriptions with detailed information about patient/sample demographics, imaging modalities, annotation standards, and multi-center data harmonization. Include explicit preprocessing and augmentation details to improve reproducibility.
	**Response:**<br>We appreciate your request for further details regarding the dataset and methodology. We have significantly expanded the 'Dataset and preprocessing' section to explicitly describe the data origins, imaging modalities, and annotation standards. Referencing the foundational work by Tschandl et al. (2018), we have clarified the multi-center nature of the dataset (sourced from Vienna and Queensland) and the harmonization steps taken to standardize images from diverse acquisition systems (e.g., digital dermatoscopy vs. digitized slides). Furthermore, we have ensured that all preprocessing steps

	(Lines: 331-341) and augmentation hyperparameters (Lines: 357-365) are described in the narrative text to facilitate exact reproducibility. Besides, to ensure exact reproducibility, we have summarized the specific data augmentation hyperparameters in a new table (Table 3).<br><br>**Changes made to the Manuscript:**<br><br>• **Lines 318–324:** We have updated the dataset description to specify the two clinical sources (Vienna and Queensland) and the three imaging modalities (MoleMax HD, DermLite, Diapositives).<br><br>• **Lines 325–329:** We have clarified the ground truth establishment (Histopathology vs. Expert Consensus).<br><br>• **Lines 365-366, 376:** We have added Table 3 to explicitly list the geometric and photometric transformation parameters used during training.
**3.**	Robust Experimental Design and Statistical Rigor. Implement stratified k-fold cross-validation or repeated random splits with detailed fold-wise metrics. Present statistical significance testing (e.g., paired t-test, Wilcoxon) for key results and detailed per-class performance breakdowns.
	**Response:**<br><br>We thank the reviewer for this important suggestion regarding methodological rigor. Our study has a specific and novel goal to perform a **controlled**, **comparative analysis of six different fusion architectures** that are explicitly designed and evaluated with **explainable AI (XAI) integration in mind.** By integrating and contrasting two major XAI techniques (Grad-CAM and SHAP), we aim to fill a critical gap in the literature, which often treats architectural performance and post-hoc explainability as separate concerns.<br><br>To ensure a fair and interpretable comparison with **existing literature** and to maintain a consistent basis for our XAI analysis, we deliberately adopted the standard one-hold-out data split of the dataset. This is the same setup used by the majority of studies we have benchmarked against in Table 5 of our manuscript.<br><br>Introducing a new k-fold validation protocol at this stage would create an unfair comparison with prior work, as performance metrics are highly sensitive to the specific data partitioning strategy. More critically, it would decouple our core findings from the established benchmark literature, undermining our ability to contextualize the contribution of our XAI-integrated architectural comparisons.
**4.**	Expanded Explainability Approaches. Integrate additional explainability frameworks (e.g., Grad-CAM, LIME, SHAP) with relevant qualitative and quantitative analyses. Include case studies explaining both successes and errors to support clinical interpretability.
	**Response:**<br><br>We thank the reviewer for suggesting that we expand our explainability analysis further. We agree that a deeper investigation into model interpretability would significantly strengthen the clinical relevance of our work. In our experiments, we have employed two major complementary XAI frameworks, Grad-CAM (which highlights salient image regions based on gradient flow) and SHAP (which quantifies feature importance based on game theory). We selected these two methods specifically because they follow different fundamental principles. Grad-CAM provides spatial visual explanations, while SHAP offers a quantitative, feature-attribution perspective. This dual-method approach provides a more robust and multifaceted view of model reasoning than using a single

	technique.<br><br>We have added a dedicated subsection titled "Diagnostic Error Analysis and Interpretability Consistency" and a new figure (Fig 13). In this subsection we conducted a granular analysis of misclassified cases from our top-performing model. We identified specific failure modes, including False Positives driven by artifact dependency (e.g., air bubbles) and False Negatives caused by low-contrast lesion boundaries. Moreover, we compared the alignment between Grad-CAM and SHAP.<br><br>**Changes made to the Manuscript:**<br><br>• **Lines 888–911:** Added the new subsection "Diagnostic Error Analysis and Interpretability Consistency" analyzing specific failure modes.<br><br>• **Figure 13:** Added a new figure illustrating a False Positive (Artifact) and a False Negative (Low Contrast) with corresponding Grad-CAM and SHAP visualizations.
**5.**	Ablation and Component Impact Analysis. Provide detailed ablations for model components, data preprocessing steps, and optimization algorithms. Report both quantitative metrics and qualitative results indicating the contribution of each component.
	**Response:**<br><br>We thank the reviewer for highlighting the importance of ablation studies to isolate the contribution of individual components. We would like to clarify that the core experimental design of our study is, in essence, **a comprehensive and systematic architectural ablation analysis.** Our primary objective is to perform a controlled, comparative evaluation of six distinct fusion architectures (our main novel component) against each other and against established CNN and Transformer baselines. Therefore, the detailed comparison presented in Section 4 (Results and Discussion), which reports quantitative metrics (F1-score, Precision, Recall) and qualitative XAI insights (Grad-CAM/SHAP visualizations) for each model, directly serves as an ablation study of the "fusion technique" variable.
**6.**	Broader Benchmarking. Benchmark against state-of-the-art deep learning methods relevant to the problem, including attention-based, transformer, and hybrid architectures. Include efficiency comparisons relevant for clinical use.
	**Response:**<br><br>Thanks. Our current experimental design was meticulously crafted to answer the primary research question "How do different fusion strategies between CNNs and ViTs affect performance and, critically, explainability in skin lesion classification?" The side-by-side comparison of six novel fusion architectures on a standardized, challenging benchmark (HAM10000) provides a clear and controlled answer. In Table 5, we have benchmarked our results against relevant recent studies. Adding extensive new comparisons against a broad array of external SOTA models, while informative, would shift the paper's focus from an in-depth architectural investigation to a general benchmarking study. This could dilute the paper's novel message. Furthermore, such comparisons are often hindered by differences in codebase, training protocols, and data splits, making direct, fair comparisons exceptionally complex and potentially misleading without a full re-implementation, which is beyond the scope of this focused contribution.
**7.**	Handling Imbalanced Data and Rare Cases. Discuss strategies employed for imbalanced data or rare conditions, reporting per-class metrics, and experiments with weighted losses or data augmentation.
	**Response:**

	We appreciate the reviewer highlighting the issue of class imbalance. In this work, we addressed the uneven distribution of skin lesion classes using a comprehensive data augmentation strategy that targets minority classes. As detailed in the Data augmentation section, we applied specific geometric and photometric transformations to the minority classes to artificially increase their representation in the training set. This approach allows the model to learn from a more diverse set of variations for rare lesion types. We have reported detailed per-class performance metrics, including Precision, Recall, and F1-score, in Table 4. These results demonstrate that our strategy was effective, as evidenced by the high performance on rare classes. For example, FM6 achieved an F1-score of 0.94 for the vascular lesion (VASC) class despite it being a minority category.
**8.**	Clinical Integration and Translation. Elaborate on considerations for real-world deployment in clinical settings, regulatory challenges, device heterogeneity, and ethical implications. Propose plans for external, multicenter, or prospective validation.
	**Response:**<br><br>We appreciate this critical feedback. We recognize that high diagnostic accuracy on a retrospective dataset is only the first step towards clinical utility. We have substantially expanded the Integration into clinical workflow section to address the complex translational barriers required for real-world deployment. Specifically, we added a detailed analysis covering three key areas. First, we discussed the challenge of device heterogeneity and the need for domain adaptation to handle images from different hardware. Second, we addressed regulatory considerations for Software as a Medical Device (SaMD), emphasizing the need for explainability to meet transparency standards. Third, we incorporated ethical implications, particularly focusing on the risk of automation bias and the necessity of keeping the human expert in the loop. Furthermore, we refined the Conclusion and future works section to explicitly prioritize external, multi-center, and prospective validation studies to prove robustness beyond the HAM10000 benchmark.<br><br>**Changes made to the Manuscript:**<br><br>• **Lines 939–953:** Added a comprehensive discussion on regulatory, technical, and ethical challenges in the "Integration into clinical workflow" section.<br><br>• **Lines 994–999:** Updated the "Conclusion and future works" section to explicitly propose prospective multi-center validation.
**9.**	Enhanced Literature Integration. Expand literature review to include recent advances and foundational works in biomedical AI, especially explicability, federated learning, and clinical translation, citing:<br><br>https://www.sciencedirect.com/science/article/pii/S1476927125000283<br>https://link.springer.com/article/10.1007/s11831-025-10255-2<br>https://www.sciencedirect.com/science/article/pii/S1476927125001604?via%3Dihub<br>https://www.nature.com/articles/s41598-025-12602-6<br>https://pubmed.ncbi.nlm.nih.gov/40751377/<br>https://www.nature.com/articles/s41598-025-11574-x<br>https://link.springer.com/article/10.1007/s11831-025-10315-7<br>https://www.nature.com/articles/s41598-025-14333-0<br>https://link.springer.com/article/10.1007/s11831-025-10376-8?utm_source=rct_congratemailt&utm_medium=email&utm_campaign=nonoa_20251002&utm_content=10.1007%2Fs11831-025-10376-8<br>https://link.springer.com/article/10.1007/s11831-025-10379-5<br>https://link.springer.com/article/10.1007/s11831-025-10414-5<br>https://link.springer.com/article/10.1007/s11831-025-10411-8

	https://www.sciencedirect.com/science/article/pii/S1746809425013631?r<br>https://journals.tubitak.gov.tr/biology/vol49/iss5/9/
	**Response:**<br><br>We thank the reviewer for providing this comprehensive list of recent and foundational works. We applied a systematic selection criterion to the suggested literature, prioritizing studies that specifically address hybrid feature fusion architectures and Explainable AI in medical imaging. Consequently, we have integrated the publications that align with these methodological scopes into our manuscript. These citations strengthen our discussion on the current state of biomedical AI and the growing necessity for transparent diagnostic tools.<br><br>**Changes made to the Manuscript:**<br><br>• **Lines 53–61:** We have cited the T-FSPANNet, DY-FSPAN, PABT-Net models in the Introduction section. These references illustrate the broader architectural shift in medical imaging towards pyramidal attention and hybrid feature fusion, thereby substantiating the design choices of our proposed framework.<br><br>• **Lines 221–230:** We have added a detailed discussion of the BCB-CSPA network in the Related Works section. We recognize this work as a key benchmark for attention-driven skin lesion classification on the HAM10000 dataset and have discussed how our ViT-Fusion approach complements this direction.
